# Effect of Dietary Sugarcane Bagasse on Reproductive Performance, Constipation, and Gut Microbiota of Gestational Sows

**DOI:** 10.3390/ani14172523

**Published:** 2024-08-30

**Authors:** Rong-Hui Huang, Bing-Bing Zhang, Juan Wang, Wei Zhao, Yu-Xuan Huang, Ying Liu, Lv-Hui Sun, Zhang-Chao Deng

**Affiliations:** 1 Key Laboratory of Smart Farming Technology for Agricultural Animals, Ministry of Agriculture and Rural Affairs, State Key Laboratory of Agricultural Microbiology, Hubei Hongshan Laboratory, Frontiers Science Center for Animal Breeding and Sustainable Production, College of Animal Science and Technology, Huazhong Agricultural University, Wuhan 430070, China; huangrh@webmail.hzau.edu.cn (R.-H.H.);; 2 Newhope Liuhe Co., Ltd., Beijing 100102, China; 3 Tianjin Animal Disease Prevention and Control Center, Tianjin 300402, China

**Keywords:** gestation sow, reproductive performance, sugarcane bagasse, short-chain fatty acids, gut microbiota

## Abstract

**Simple Summary:**

Sugarcane bagasse (SB), one of the more unconventional feedstuffs, is a processed agricultural and industrial by product. Our results show that SB contains higher levels of crude fiber (42.1%) and neutral detergent fiber (81.3%) than soybean hulls, and it also exhibited the highest volumetric expansion when soaked in water (50 g expanding to 389.8 mL) compared to the other six materials we tested (vegetable scraps, soybean hulls, wheat bran, rice bran meal, rice bran, and corn DDGS). Meanwhile, previous studies have reported that SB is worth being developed and utilized as a pig feedstuff, which could effectively alleviate the shortage of feed resources in the swine industry. In the present study, we explored the effects of SB on the reproductive performance, constipation, and gut microbiota of gestational sows. Our results showed that dietary supplementation of 5% SB can be used as an equivalent substitute for soybean hulls to improve the reproductive performance of sows without affecting their gut microbiota.

**Abstract:**

This experiment aimed to evaluate the effects of using sugarcane bagasse (SB) as a substitute for soybean hulls and wheat bran in the diet of pregnant sows on their reproductive performance and gut microbiota. A total of seventy-two primiparous sows were randomly divided into four treatment groups, with eighteen replicates of one sow each. The sows were fed a basal diet supplemented with 0% (CON), 5%, 10%, and 15% SB to replace soybean hulls from day 57 of gestation until the day of the end of the gestation period. The results showed that SB contains higher levels of crude fiber (42.1%) and neutral detergent fiber (81.3%) than soybean hulls, and it also exhibited the highest volumetric expansion when soaked in water (50 g expanding to 389.8 mL) compared to the other six materials we tested (vegetable scraps, soybean hulls, wheat bran, rice bran meal, rice bran, and corn DDGS). Compared with the CON, 5% SB significantly increased the litter birth weight of piglets. Meanwhile, 10% and 15% SB significantly increased the rates of constipation and reduced the contents of isobutyric acid and isovaleric acid in feces. Furthermore, 10% and 15% SB significantly disturbed gut microbial diversity with increasing *Streptococcus* and decreasing *Prevotellaceae_NK3B31-group* and *Christensenellaceae_R-7-group* genera in feces. Interestingly, *Streptococcus* had a significant negative correlation with isobutyric acid, isovaleric acid, and fecal score, while *Prevotellaceae_NK3B31-group* and *Christensenellaceae_R-7-group* had a positive correlation with them. In conclusion, our study indicates that 5% SB can be used as an equivalent substitute for soybean hulls to improve the reproductive performance of sows without affecting their gut microbiota.

## 1. Introduction

Food security, shortages of feed resources, and food competition between humans and animals have become more and more serious in recent years [1,2]. Conventional feed for livestock primarily consists of grain feed, which makes up over 80% of the total composition. However, the extensive use of corn and soybean meal as feed increases the risk of food security and is not conducive to the sustainable development of animal husbandry [3]. Notably, unconventional feed ingredients refer to feed ingredients that are less used in feed formulations or those for which there is a lack of understanding about their nutritional characteristics and feeding value [4]. They are mainly derived from agricultural by products and food industry by products, and 70% of food processing by products can be used as feed [5]. Although unconventional feed ingredients have the characteristics of poor palatability, containing a variety of anti-nutritional factors, and low feeding value, they have a wide range of sources, many types, large total amounts, and are rich in nutrients and bioactive components [6]. Previous studies have shown that several unconventional feed resources, such as azolla pinnata, gardenia pomace, and sugarcane molasses, can be used to replace conventional feed ingredients and have the potential to improve the production performance, gut health, and meat quality of livestock and poultry [7,8,9]. Therefore, developing and utilizing novel unconventional food resources is a feasible strategy for reducing conventional feed ingredients, which is of great significance for livestock husbandry and food security.

Gestational sows undergo complex physiological changes, and restricted feeding is widely adopted to maintain their normal reproductive performance [10,11]. However, the restricted feeding and increased uterus volume slow down gastrointestinal motility, causing a prolonged retention of feces in the colon and physiological constipation in late pregnant sows [12,13]. Previous studies have shown that about 64.6% of pregnant sows experienced moderate to severe constipation that negatively affected their reproductive performance [14]. Notably, constipation can elevate bacterial endotoxin levels in the gastrointestinal tract, leading to intestinal nervous system disorders and postpartum galactosis syndrome [15]. Moreover, the gut dysbiosis caused by constipation during the parturition process can aggravate oxidative stress and disrupt metabolic homeostasis, which seriously endangers the health of sows and their offspring [12,13,14]. 

Sugarcane bagasse (SB), a fiber source, is a processed agricultural and industrial by product, exhibiting a low protein and high fiber content when compared to other unconventional feedstuffs [16]. A previous study reported that dietary supplementation with fermented SB not only improved the growth performance of fattening pigs, but also reduced feed consumption and feeding costs [17]. These findings suggested that SB is worth being developed and utilized as a pig feedstuff, which could effectively alleviate the shortage of feed resources in the swine industry. Nevertheless, SB is a valuable food resource, and its effects on pregnant sows are not well understood. Therefore, this study aims to evaluate the effects of SB on the reproductive performance, constipation, and gut microbiota of gestational sows. 

## 2. Materials and Methods

### 2.1. Animals, Experimental Design, and Sample Collection 

The animal protocol was approved by the Institutional Animal Care and Use Committee of Huazhong Agricultural University, China. A total of 64 primiparous sows (DanBred × Landrace) with similar body conditions were randomly divided into 4 treatments, with 16 replicates of 1 sow each [18]. The sows form the four groups were fed a basal diet (Table 1) supplemented with 0% (CON), 5%, 10%, and 15% SB to replace soybean hulls from day 57 of gestation until the end of the gestation period. The sows were fed following the standard protocol of the farm in the individual gestation stall, and water was given ad libitum following the standard management regulations and normal immunization procedures at the farm during the experimental period. Notably, due to the high constipation rate of sows in the 15% SB group, the feed in the 15% SB was temporarily replaced with 5% SB at 103 days of pregnancy. At day 100 of gestation, 8 sows from each group were randomly selected to collect fresh fecal samples for subsequent short-chain fatty acids (SCFAs) and microbial analysis [19,20,21]. After farrowing, the litter size, total number of piglets born alive, stillborn, mummies, healthy piglets, weak piglets, and the birth weights of the offspring were recorded. 

### 2.2. Determination of Nutrients and Swelling Rates of Different Fiber Ingredients 

The moisture, crude ash (Ash), crude protein (CP), crude fiber (CF), ether extract (EE), and neutral detergent fiber (NDF) contents in the soybean hulls, wheat bran, and sugarcane bagasse were measured as previously described [23]. Seven feed ingredients (wheat bran, corn DDGS, rice bran meal, rice bran, soybean hulls, SB, and vegetable scraps) with high crude fiber contents were selected for the determination of swelling rates [24]. The feed ingredients were pulverized, measured to 100 mL volume, and then poured into a beaker and weighed. A total of 100 mL warm water at 37 °C was added to each beaker and stirred with a glass rod. Subsequently, 100 mL of warm water was added to each beaker again, and after the expansion of the ingredients stabilized (with a noticeable water layer), the corresponding graduations for each ingredient were recorded. The volume of the ingredient expansion was converted into expansion volume per unit ingredient weight, and the expansion volumes of different materials were compared by sorting. 

### 2.3. Determination of Fecal Score and Constipation Rate during Pregnancy

Fecal scoring was conducted on sow feces in the morning and afternoon each day during the experimental period. The fecal score after defecation was evaluated by visual qualitative method as previously described [25]. Each score represent as follow: a score of 1 indicates hard, dry, and pellet-shaped; a score of 2 indicates firm and formed stool; a score of 3 indicates moist and soft stool that retains its shape; a score of 4 indicates soft but unformed stool that assumes the shape of its container; and a score of 5 indicates watery liquid that can be poured [25]. Stools that are hard, dry, pellet-shaped, and firm, with a low water content, are often indicative of constipation. Therefore, a fecal score less than 3 is considered to be indicative of constipation. 

### 2.4. Determination of the Fecal SCFAs Profile 

The contents of the fecal SCFAs were determined by gas chromatography as previously described [26]. In brief, 1 g of feces was mixed with 1 mL of chromatography-grade methanol and vortexed for 30 s. Subsequently, the samples were centrifuged at 12,000× *g* for 10 min at 4 °C. The supernatants were diluted with 25% metaphosphoric acid (*v*:*v*, 1:5), filtered through a 0.22 µm membrane, and subjected to the gas chromatography system for SCFAs measurement.

### 2.5. 16S rRNA Gene Sequencing and Microbiome Analysis

The fecal microbiota was analyzed as previously described [27,28]. In brief, the total microbial DNA was extracted from fecal digesta using the DNA stool mini kit (Tiangen, Beijing, China), and DNA quantity and quality were determined using a Nanodrop spectrophotometer. Subsequently, the high-quality DNA was amplified with the primers 338F (5′-ACTCCTACGGGAGGCAGCA-3′) and 806R (5′-GGACTACHVGGGTWVTAAT-3′). All purified amplicons were sequenced using an Illumina MiSeq/NovaSeq platform (Illumina, San Diego, CA, USA) following the standard protocols [29]. The microbiota composition and alpha diversity were determined, and a principal coordinate analysis (PCoA) based on Bray–Curtis distances was carried out, using the OmicStudio tools [30,31]. A linear discriminant analysis (LDA) effect size (LEfSe) analysis with LDA score > 3 was performed to identify differential microbial taxonomic between groups [32]. Correlation analysis between differential genera and different indices was carried out using the Spearman’s rho correlation test [33], and correlations with coefficients < −0.3 or >0.3 and false discovery rate (FDR)-adjusted *p*-values < 0.05 were considered significant correlation. The Kruskal–Wallis test was performed to calculated significant differences, and the Benjamani–Hochberg method was used to adjust *p*-values. 

### 2.6. Statistical Analysis

Statistical analyses were performed using SPSS version 19.0 (Chicago, IL, USA). Data are presented as mean ± SD. Levene’s test of homogeneity of variance was used to evaluate unequal variances before ANOVA analysis. Data were analyzed by one-way ANOVA, with a significance level of *p* < 0.05. 

## 3. Results

### 3.1. Nutrients of Different Fiber Raw Materials

The nutrients in the soybean hulls, wheat bran, and SB are presented in Table 2. The levels of Ash, CP, and EE in the SB were 3.4%, 1.38%, and 0.9%, respectively, which are lower than those in soybean hulls and wheat bran. Additionally, SB also has the lowest NE level of the three raw materials at 1000 Kcal/Kg. However, SB contains higher levels of CF and NDF than soybean hulls and wheat bran at 42.1% and 81.3%, respectively. 

### 3.2. Water Swelling Performance of Different Fiber Raw Materials

**Figure 1:**
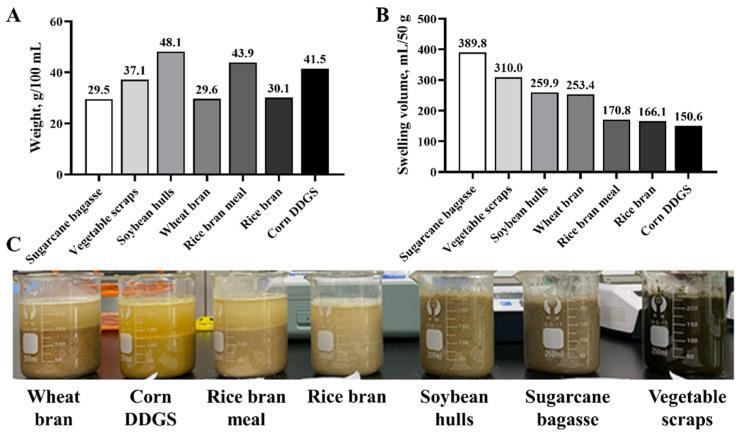
The weight (**A**) of seven different fiber ingredients at the volume of 100 mL. The swelling volume (**B**) of seven different fiber ingredients at the weight of 50 g. Image of seven different fiber ingredients after by absorbing water (**C**). DDGS, distillers’ dried grains with soluble.

The water swelling performance results of the different fiber raw materials are shown in Figure 1. Among these seven tested materials, SB exhibited the lightest weight (29.5 g) at the same volume of 100 mL (Figure 1A), and the highest volumetric expansion when soaked in water (50 g expanding to 389.8 mL; Figure 1B). Meanwhile, the swelling performance of the seven materials in water is as follows: SB > vegetable scraps > soybean hulls > wheat bran > rice bran meal > rice bran > corn DDGS (Figure 1B). Image of the seven different fiber ingredients after absorbing water are shown below (Figure 1C). 

### 3.3. Reproductive Performance and Fecal Parameters of Sows

**Figure 2:**
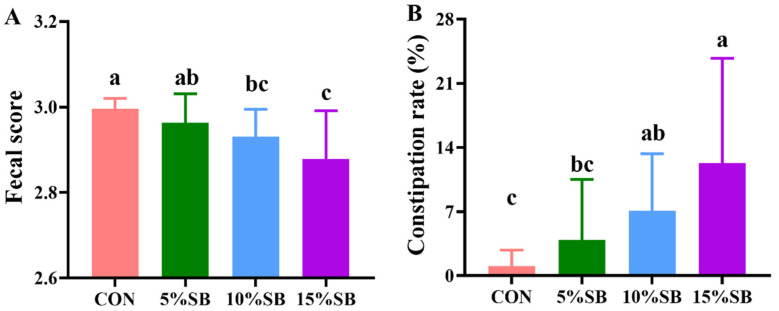
Effect of SB on fecal score (**A**) and constipation rate (**B**) of sows. Data are presented as mean ±SD, n = 16. Labeled means in a row with different letters differ, *p* < 0.05. The different colors represent different treatment groups, in which red represents CON group, green represents 5%SB group, blue represents 10%SB group, and purple represents 15%SB group. CON, control group; SB, sugarcane bagasse group.

The reproductive performance results of the sows are shown in Table 3. Compared to the CON, 5% SB significantly increased (*p* < 0.05) the litter birth weight by 10.3%. Expect for this, no significant differences (*p* > 0.05) were found in litter size, the number of piglets born alive, healthy piglets, weak piglets, stillborn, mummies, and birth weight/piglet among the four groups. Meanwhile, compared to the CON, both 10% and 15% SB groups significantly reduced (*p* < 0.05) the fecal score, and increased (*p* < 0.05) the constipation rate of pregnant sows (Figure 2). Moreover, 5% SB significantly increased (*p* < 0.05) the days of gestation compared to 15% SB. Notably, 5% SB had no effect (*p* > 0.05) on the fecal score and constipation rate of pregnant sows compared to the CON (Figure 2). 

### 3.4. Short-Chain Fatty Acids Status 

**Figure 3:**
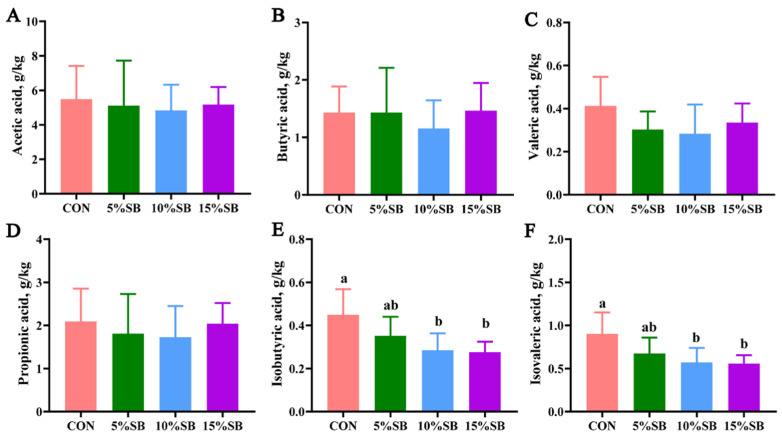
Effect of SB on short-chain fatty acids in the feces of sows. Data are presented as mean ± SD, n = 8. Labeled means in a row with different letters differ, *p* < 0.05. The different colors represent different treatment groups, in which red represents CON group, green represents 5%SB group, blue represents 10%SB group, and purple represents 15%SB group. CON, control group; SB, sugarcane bagasse group.

Compared to the CON, no significant differences (*p* > 0.05) were found in acetic acid, propionic acid, butyric acid, and valeric acid among the four groups (Figure 3A–D). However, both the 10% SB and 15% SB groups showed reduced (*p* < 0.05) levels of isobutyric acid and isovaleric acid in the feces of sows by 36.7–38.5% compared with the CON (Figure 3E,F). 

### 3.5. Gut Microbiota

**Figure 4:**
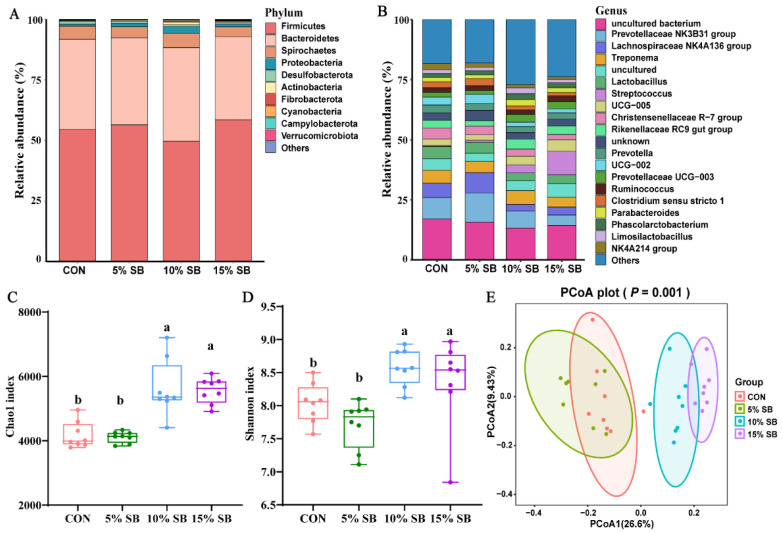
Composition and diversities of the fecal microbiota in sows. Relative abundance of the top 10 phyla (**A**) and top 20 genera (**B**) in the four groups. Differences in alpha diversities were calculated using the Chao1 index (**C**) and Shannon index (**D**), respectively. Principal coordinates analysis (PCoA) plots were generated using Bray–Curtis distances (**E**). n = 8. Labeled means with different superscript letters are significantly different, *p* < 0.05. CON, control group; SB, sugarcane bagasse group.

**Figure 5:**
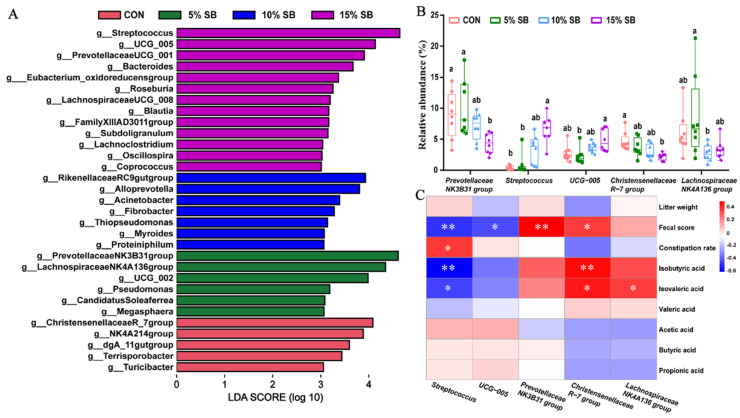
Differential enrichment of the bacterial taxa was identified using LEfSe with an LDA score > 3.0 (**A**). Relative abundance of five differentially enriched bacterial features (**B**). Spearman correlation between fecal score and short–chain fatty acid and the differentially expressed genus (**C**). n = 8. Labeled means with different superscript letters are significantly different, *p* < 0.05. Correlation coefficients and *p*–values were determined via Spearman’s rho correlation test. *, *p* < 0.05, **, *p* < 0.01. LDA, Linear discriminant analysis; LEfSe, LDA effect Size; CON, control group; SB, sugarcane bagasse group.

The effects of SB on gut microbiota are shown in Figure 4 and Figure 5. A total of 32 fecal samples from the sows were subjected to 16S rRNA gene sequencing, and a total of 15,356 operational taxonomic units (OTUs) were observed (Supplementary Table S1). All the qualified sequences were then assigned to 30 phyla and 594 genera (Supplementary Tables S2 and S3). Of them, the top 10 phyla were predominantly identified as follows: Firmicutes, Bacteroidetes, Spirochaetes, Proteobacteria, Desulfobacterota, Actinobacteria, Fibrobacterota, Cyanobacteria, Campylobacterota, and Verrucomicrobiota (Figure 4A), and the top 20 genera are shown in Figure 4B. The alpha diversity results showed that both the 10% and 15% SB groups had a higher (*p* < 0.05) Chao1 index and Shannon index than those of the CON and 5% SB groups (Figure 4C,D). Meanwhile, a further PCoA analysis showed that both the 10% and 15% SB groups were significantly separated (*p* = 0.001) from the CON and 5% groups (Figure 4E). Notably, no significant differences in microbial diversity were observed between the 5% SB and CON groups (Figure 4C–E). 

Moreover, LEfSe analysis was performed to identify differential bacterial taxa among the four groups, and the results revealed that a total of 31 discriminative bacterial taxa (including five genera in the CON group, six genera in the 5% SB group, seven genera in the 10% SB group, and thirteen genera in the 5% SB group) were identified at genus level (Figure 5A). Among these differential genera, the top 5 differential genera are as follows: *Streptococcus*, *Prevotellaceae_NK3B31-group*, *Lachnospiraceae_NK4A136-group*, *Christensenellaceae_R-7-group*, and *UCG-005* (Figure 5B). Notably, the 5% SB had no effect (*p* > 0.05) on the abundance of these five genera when compared to the CON group (Figure 5B). However, 10% and (or) 15% SB groups showed reduced (*p* < 0.05) abundances of *Prevotellaceae_NK3B31-group* and *Christensenellaceae_R-7-group*, and increased (*p* < 0.05) abundances of *Streptococcus*, when compared to the CON and (or) 5% SB groups (Figure 5B). A further spearman correlation analysis showed that *Streptococcus* had a negative correlation (R < −0.44, *p* < 0.05) with isobutyric acid, isovaleric acid, and fecal score, while it had a positive correlation (R > 0.38, *p* < 0.05) with the constipation rate (Figure 5C, Supplemental Table S4). The *Christensenellaceae_R-7-group* had a positive correlation (R > 0.37, *p* < 0.05) with isobutyric acid, isovaleric acid, and fecal score (Figure 5C, Supplemental Table S4). In addition, the fecal score also showed a negative correlation (R < −0.43, *p* < 0.05) with *UCG-005*, and a positive correlation (R > 0.52, *p* < 0.01) with *Prevotellaceae_NK3B31-group* (Figure 5C, Supplemental Table S4). 

## 4. Discussion

Dietary fibers are an important nutrient in sows’ diets, and a moderate supplementation of dietary fiber in the diet of pregnant sow could regulate multiple biological functions [34]. The current study shows that dietary supplementation of 5% SB to replace soybean hulls can improve the reproductive performance of pregnant sows. Specifically, 5% SB increased the litter birth weight of piglets. Meanwhile, the nutrition composition analysis indicated that SB contains higher levels of CF and NDF than soybean hulls and wheat bran at 42.1% and 81.3%, respectively. These results are similar to previous studies, which have reported that dietary fibers, such as SB, inulin, and bamboo powder, can improve the reproductive performance of sows [35,36,37]. Appropriately increasing fiber levels can enhance satiety in sows, reduce stereotypical behaviors, alleviate sow stress, and improve reproductive performance to a certain extent [38,39]. However, our study found that over 10% SB supplementation did not affect the reproductive performance of sows; instead, it increased the incidence of constipation in sows. These results could be due to the special and different chemical structure of different dietary fibers, resulting in differences in their function such as water absorption, swelling, viscosity, and fermentability [40,41]. In this study, we found that SB exhibited the highest volumetric expansion when soaked in water among of seven tested materials. This result suggested that SB have greatly swelling performance, which could increase the water content of intestinal chyme and fecal lubrication [42]. Notably, the polysaccharide molecules in dietary fibers can thicken after water absorption and hydrate in a concentration-dependent manner, forming intertwined gel-like structures, which will increase chyme viscosity, reduce gastrointestinal emptying rates, and cause constipation [43,44]. Overall, dietary supplementation with high doses of SB over 10% might increase the water content of intestinal chyme, and subsequently increase chyme viscosity due to polysaccharide molecules, which could explain why over 10% SB leads to constipation in gestational sows [13,45]. 

Previous studies have discovered that dietary fibers can be fermented by gut microbes to produce SCFAs, such as acetic, propionic, butyric, isovaleric, and isobutyric acid [39]. These SCFAs have functions such as providing energy, anti-inflammatory, antioxidant, and hormone regulation, which can regulate the host physiology [46,47,48]. In this study, our results showed that dietary supplementation of over 10% SB reduced the levels of isovaleric and isobutyric acid in feces of sows, while acetic, propionic, and butyric acid were not affected by SB treatment. A previous study showed that isobutyric acid and isovaleric acid are the main products of protein fermentation and increasing the content of fermentable fiber could reduce these two acids [49]. Therefore, the changes in SCFAs profiling may owe to SB having a higher crude fiber content than soybean hulls, which alters gut microbial composition in the hindgut, thereby shifting from protein fermentation to fiber fermentation [50]. As expected, our microbial results showed that over 10% SB supplementation in the diet markedly altered the gut microbial diversity, as evident by the increased alpha diversity and differential beta diversity. Meanwhile, we also found that both isobutyric acid and isovaleric acid had a negative correlation with *Streptococcus*, while had a positive correlation with *Christensenellaceae_R-7-group*. Taken together, these outcomes implied that the reduction in isobutyric and isovaleric acid content may be due to the fact that high doses of SB supplementation ≥ 10% alter the sow gut microbiota, especially *Streptococcus* and *Christensenellaceae_R-7-group*. 

Moreover, dietary fibers are important energy sources for hindgut-residing microbiota, which interact directly with gut microbes and lead to produce postbiotics that can stimulate mucus secretion and improve host intestinal health [51,52,53]. Due to their significant effects on the gut microbiome [34], 16S rRNA gene sequencing technology was utilized to analysis the sow gut microbiota after SB treatment. Compared with the CON, 10% and 15% SB reduced the genera of *Prevotellaceae_NK3B31-group* and *Christensenellaceae_R-7-group*, while 5% SB did not. *Prevotellaceae_NK3B31-group* is closely associated with gut health, which can competitively inhibit the growth of pathogenic bacteria, maintain gut microbiota balance, and support normal immune system function [54]. Moreover, *Christensenellaceae_R-7-group* is believed to contribute to maintaining a balanced gut microbiota by promoting the growth of beneficial bacteria and inhibiting the proliferation of harmful microbes [55]. These outcomes suggest that high doses of SB supplementation in the diets of sows may disrupt gut microbiota balance, potentially exerting adverse effects on gut health of gestational sows [40]. Interestingly, our results found that the abundance of *Prevotellaceae_NK3B31-group* and *Christensenellaceae_R-7-group* are positively linked to fecal score, suggesting that members of these two genera may contribute to constipation of gestational sows [56]. On the other hand, *Streptococcus* genus is a type of lactic acid bacteria [57]. They can ferment dietary fiber and yield SCFAs, which helps maintain the acid–base balance in gut and provides energy for intestinal cells [58]. In this study, we found that the abundance of *Streptococcus* increased with increased SB supplementation, which might be due to that SB increased the dietary fiber available for *Streptococcus* growth [34]. Meanwhile, our results showed that the *Streptococcus* genus had a negative correlation with fecal score, but had a positive correlation with isobutyric acid, isovaleric acid, and constipation rate. These outcomes suggested that the members of this genus may play important roles in regulating constipation in gestational sows due to isobutyric and isovaleric acid [59,60], which needs to be further explored. Moreover, due to the unclear functionality of the *UCG-005* genus, the effects of changes in this bacterium due to SB treatment remain unknow. Thus, it is hard for us to explain why *UCG005* was increased in the 15% SB treatment group, and its roles in sow’s gut health need to be further explored. 

## 5. Conclusions

In summary, this study indicates that 5% SB supplementation in the diet of gestational sows can improve the litter birth weight of piglets without disrupting the gut microbiota homeostasis of sows. Therefore, replacing an equivalent amount of soybean hulls with 5% SB in the diet of gestational sows is a feasible alternative.

## Figures and Tables

**Table 1:** Ingredients and nutrient composition of the experimental diets.

Items	CON	5% SB	10% SB	15% SB
Corn	42.195	43.045	44.005	44.875
Soybean meal	7.7	9.5	11.2	13.0
Wheat bran	22.9	20.2	17.5	14.8
Soybean hulls	15	10	5	0
Sugarcane bagasse	0	5	10	15
Flour	8	8	8	8
Stone powder	1.22	1.20	1.16	1.12
Soybean oil	1.0	1.0	1.0	1.0
NaCl	0.34	0.34	0.35	0.35
CaHPO_4_	0.30	0.42	0.56	0.68
L-Lysine H_2_SO_4_	0.27	0.23	0.19	0.15
NaHCO_3_	0.20	0.20	0.20	0.20
Mineral premix ^1^	0.20	0.20	0.20	0.20
MgO	0.20	0.20	0.20	0.20
Choline chloride	0.10	0.10	0.10	0.10
Antifungal agent	0.10	0.10	0.10	0.10
L-Threonine	0.09	0.08	0.06	0.05
DL-Methionine	0.05	0.05	0.04	0.04
Vitamin premix ^2^	0.05	0.05	0.05	0.05
Antioxidants	0.04	0.04	0.04	0.04
Xylanase	0.03	0.03	0.03	0.03
Phytase	0.015	0.015	0.015	0.015
Total (%)	100	100	100	100
Nutrient levels ^3^				
NE (MJ/kg)	9.21	9.21	9.21	9.21
DM, %	88.49	88.63	88.77	88.91
CP, %	12.98	13.01	12.99	13.01
Ash, %	5.03	5.03	5.03	5.00
CF, %	8.83	9.05	9.27	9.49
NDF, %	22.44	22.63	22.82	23.02
ADF, %	10.38	10.64	10.89	11.15
Ca, %	0.70	0.70	0.70	0.70
TP, %	0.50	0.50	0.50	0.50
SID Lys, %	0.60	0.60	0.60	0.60
SID Met, %	0.22	0.22	0.22	0.22
SID Thr, %	0.43	0.43	0.42	0.42

^1^ Mineral premix provided per kilogram of diet: Zn 100 mg, Fe 100 mg, Cu 6 mg, Mn 4 mg, I 0.14 mg, Se 0.3 mg. ^2^ Vitamin premix provided per kilogram of diet: VA 2200 IU, VD3 220 IU, VE 16IU, VK3 0.5 mg, VB6 7 mg, VB12 17.5 μg, thiamine 1.0 mg, riboflavin 3.5 mg, niacin 30 mg, pantothenic acid 10 mg, biotin 0.05 mg. ^3^ Nutrient levels were a calculated value. A regress equation NE = 7.968 + 0.28 × CP + 0.607 × EE − 0.782 × Ash − 0.05 × hemicellulose was applied [22]. NE, net energy; ME, metabolizable energy; HI, heat increment; DM, dry matter; CP, crude protein; Ash, crude ash; CF, crude fiber; NDF, neutral detergent fiber; ADF, acid detergent fiber; TP, total phosphorus; SID, standard ileal digestibility; SB, Sugarcane bagasse.

**Table 2:** The nutrients (dry matter basis) in different fibrous raw materials ^1^.

Nutritional Indicators	Soybean Hulls	Wheat Bran	Sugarcane Bagasse
DM, %	91.5	86.35	94.1
Ash, %	4.25	5.52	3.4
CP, %	9.5	15.8	1.38
EE, %	2.07	2.95	0.9
CF, %	37.5	10.3	42.1
NDF, %	60	40	81.3
NE, Kcal/Kg	1397	1492	1000

^1^ NE was a calculated value. DM, dry matter; Ash, crude ash; CP, crude protein; EE, ether extract; CF, crude fiber; NDF, neutral detergent fiber; NE, net energy; SB, Sugarcane bagasse.

**Table 3:** Effect of sugarcane bagasse on the reproductive performance of sows ^1^.

Items	CON	5% SB	10% SB	15% SB	*p*-Value
Back fat, mm	14.23 ± 1.54	14.10 ± 1.84	14.20 ± 1.49	14.18 ± 1.45	0.996
Days of gestation, d	116.3 ± 1.10 ^ab^	116.9 ± 0.60 ^a^	116.4 ± 1.30 ^ab^	115.9 ± 1.20 ^b^	0.054
Feed intake, kg	2.39 ± 0.18	2.40 ± 0.14	2.48 ± 0.19	2.48 ± 0.14	0.274
Litter size, n	14.25 ± 2.43	14.63 ± 2.03	13.43 ± 2.61	14.94 ± 2.74	0.356
Born alive, n	13.31 ± 2.27	14.06 ± 1.95	12.75 ± 2.46	13.68 ± 2.60	0.436
Healthy piglets, n	11.25 ± 2.59	12.18 ± 2.17	10.88 ± 2.33	11.94 ± 2.89	0.278
Weak piglets, n	2.06 ± 2.52	1.88 ± 3.05	1.88 ± 2.58	1.75 ± 2.38	0.990
Stillborn, n	0.75 ± 1.18	0.50 ± 0.97	0.63 ± 1.02	1.06 ± 0.93	0.457
Mummies, n	0.19 ± 0.40	0.06 ± 0.25	0.06 ± 0.25	0.19 ± 0.40	0.532
Litter birth weight, kg	15.83 ± 2.61 ^b^	17.46 ± 1.76 ^a^	15.12 ± 2.15 ^b^	16.71 ± 2.10 ^ab^	0.036
Birth weight/piglet, kg	1.21 ± 0.27	1.26 ± 0.20	1.21 ± 0.22	1.22 ± 0.19	0.933

^1^ Values are means ± SD, n = 16. Different letters indicate significant difference. CON, control group; SB, sugarcane bagasse group.

## Data Availability

Data are contained within the article and Supplemental Information files.

## Supplementary Materials

The following supporting information can be downloaded at: https://www.mdpi.com/article/10.3390/ani14172523/s1, Table S1: Operational taxonomic units (OTUs) detected by 16S rRNA gene sequencing; Table S2: Phylum level composition of bacteria among four groups; Table S3: Genus level composition of bacteria among four groups; Table S4: Differential microbes associated with different parameters by Spearman correlation analysis.

## References

[1] Fears R., ter Meulen V., von Braun J. (2019). Global food and nutrition security needs more and new science. Sci. Adv..

[2] Beal T., Gardner C.D., Herrero M., Iannotti L.L., Merbold L., Nordhagen S., Mottet A. (2023). Friend or foe? The role of animal-source foods in healthy and environmentally sustainable diets. J. Nutr..

[3] Li Z.C., Li Y.K., Lv Z.Q., Liu H., Zhao J.B., Noblet J., Wang F.L., Lai C.H., Li D.F. (2017). Net energy of corn, soybean meal and rapeseed meal in growing pigs. J. Anim. Sci. Biotechnol..

[4] Rakita S., Kokic B., Manoni M., Mazzoleni S., Lin P., Luciano A., Ottoboni M., Cheli F., Pinotti L. (2023). Cold-pressed oilseed cakes as alternative and sustainable feed ingredients: A Review. Foods.

[5] Omar A.E., Al-Khalaifah H.S., Ismail T.A., Abd El-Aziz R.M., El-Mandrawy S.A.M., Shalaby S.I., Ibrahim D. (2021). Performance, serum biochemical and immunological parameters, and digestive enzyme and intestinal barrier-related gene expression of broiler chickens fed fermented fava bean by-products as a substitute for conventional feed. Front. Vet. Sci..

[6] Kanengoni A.T., Chimonyo M., Ndimba B.K., Dzama K. (2015). Potential of using maize cobs in pig diets: A Review. Asian-Australas. J. Anim. Sci..

[7] Hassanein H.A.M., Maggiolino A., El-Fadel M.A., Palo P.D., El-Sanafawy H.A., Hussein A.M., Salem A.Z.M. (2023). Inclusion of as an unconventional feed of Zaraibi dairy goats, and effects on milk production and offspring performance. Front. Vet. Sci..

[8] Zou S., Sun C.C., Li F., Xie Y.J., Liang T., Yang Y.Q., Shi B.M., Ma Q.Q., Shi Z., Chai S. (2022). Effect of gardenia pomace supplementation on growth performance, blood metabolites, immune and antioxidant indices, and meat quality in xiangcun pigs. Animals.

[9] Xandé X., Archimède H., Gourdine J.L., Anais C., Renaudeau D. (2010). Effects of the level of sugarcane molasses on growth and carcass performance of Caribbean growing pigs reared under a ground sugarcane stalks feeding system. Trop. Anim. Health Prod..

[10] Theil P.K., Farmer C., Feyera T. (2022). Review: Physiology and nutrition of late gestating and transition sows. J. Anim. Sci..

[11] Ha S.H., Choi Y.H., Mun J.Y., Park S.R., Kinara E., Park H.J., Hong J.S., Kim Y.M., Kim J.S. (2024). Correlation between reproductive performance and sow body weight change during gestation. J. Anim. Sci. Technol..

[12] Ma T., Huang W.Q., Li Y.L., Jin H., Kwok L.Y., Sun Z.H., Zhang H.P. (2023). Probiotics alleviate constipation and inflammation in late gestating and lactating sows. Npj Biofilms Microbiomes.

[13] Lu D.D., Pi Y., Ye H., Wu Y.J., Bai Y., Lian S., Han D.D., Ni D.J., Zou X.H., Zhao J.B. (2022). Consumption of dietary fiber with different physicochemical properties during late pregnancy alters the gut microbiota and relieves constipation in sow model. Nutrients.

[14] Yu X.R., Fu C.S., Cui Z.C., Chen G.Y., Xu Y.L., Yang C.M. (2021). Inulin and isomalto-oligosaccharide alleviate constipation and improve reproductive performance by modulating motility-related hormones, short-chain fatty acids, and feces microflora in pregnant sows. J. Anim. Sci..

[15] Dimidi E., Christodoulides S., Scott S.M., Whelan K. (2017). Mechanisms of action of probiotics and the gastrointestinal microbiota on gut motility and constipation. Adv. Nutr..

[16] de Almeida G.A.P., Ferreira M.D., Silva J.D., Chagas J.C.C., Véras A.S.C., de Barros L.J.A., de Almeida G.L.P. (2018). Sugarcane bagasse as exclusive roughage for dairy cows in smallholder livestock system. Asian-Australas. J. Anim. Sci..

[17] Alokika A., Kumar A., Kumar V., Singh B. (2021). Cellulosic and hemicellulosic fractions of sugarcane bagasse: Potential, challenges and future perspective. Int. J. Biol. Macromol..

[18] Wang S.Q., Peng Z., Sun H., Han Y.M., Zhang B., Pineda L., Boerboom G., Sun L.H., Liu Y., Deng Z.C. (2024). Evaluating the impact of an organic trace mineral mix on the redox homeostasis, immunity, and performance of sows and their offspring. Biol. Trace Elem. Res..

[19] Huang W., Ma T., Liu Y., Kwok L.-Y., Li Y., Jin H., Zhao F., Shen X., Shi X., Sun Z. (2022). Spraying compound probiotics improves growth performance and immunity and modulates gut microbiota and blood metabolites of suckling piglets. Sci. China Life Sci..

[20] Wang D., Kuang Y., Lv Q., Xie W., Xu X., Zhu H., Zhang Y., Cong X., Cheng S., Liu Y. (2023). Selenium-enriched Cardamine violifolia protects against sepsis-induced intestinal injury by regulating mitochondrial fusion in weaned pigs. Sci. China Life Sci..

[21] Eudy B.J., Odle J., Lin X., Maltecca C., Walter K.R., McNulty N.P., Fellner V., Jacobi S.K. (2023). Dietary prebiotic oligosaccharides and arachidonate alter the fecal microbiota and mucosal lipid composition of suckling pigs. J. Nutr..

[22] He C., Xu S., Li Z., Yu Z., Levesque C., Zhang Y., Wang Z., Shi C., Wang F., Liu H. (2024). Determination and prediction of the net energy content of wheat bran for pregnant sow. Arch. Anim. Nutr..

[23] Latimer G.W. (2023). Official Methods of Analysis of AOAC International.

[24] Hua M., Lu J., Qu D., Liu C., Zhang L., Li S., Chen J., Sun Y. (2019). Structure, physicochemical properties and adsorption function of insoluble dietary fiber from ginseng residue: A potential functional ingredient. Food Chem..

[25] Lan R.X., Li T.S., Kim I. (2017). Effects of xylanase supplementation on growth performance, nutrient digestibility, blood parameters, fecal microbiota, fecal score and fecal noxious gas emission of weaning pigs fed corn-soybean meal-based diet. Anim. Sci. J..

[26] Li Y., He J., Zhang L., Liu H., Cao M., Lin Y., Xu S., Che L., Fang Z., Feng B. (2024). Improvement of insulin sensitivity by dietary fiber consumption during late pregnant sows is associated with gut microbiota regulation of tryptophan metabolism. Anim. Microbiome.

[27] Deng Z.C., Yang J.C., Huang Y.X., Zhao L., Zheng J.S., Xu Q.B., Guan L.L., Sun L.H. (2023). Translocation of gut microbes to epididymal white adipose tissue drives lipid metabolism disorder under heat stress. Sci. China Life Sci..

[28] Deng Z.C., Wang J., Wang J., Yan Y.Q., Huang Y.X., Chen C.Q., Sun L.h., Liu M. (2024). Tannic acid extracted from gallnut improves intestinal health with regulation of redox homeostasis and gut microbiota of weaned piglets. Anim. Res. One Health.

[29] Naik T., Sharda M.C.P.L., Virbhadra K., Pandit A. (2023). High-quality single amplicon sequencing method for illumina MiSeq platform using pool of ‘N’ (0-10) spacer-linked target specific primers without PhiX spike-in. BMC Genom..

[30] Cao K.X., Deng Z.C., Liu M., Huang Y.X., Yang J.C., Sun L.H. (2023). Heat stress impairs male reproductive system with potential disruption of retinol metabolism and microbial balance in the testis of mice. J. Nutr..

[31] Yang J.C., Huang Y.X., Sun H., Liu M., Zhao L., Sun L.H. (2023). Selenium deficiency dysregulates one-carbon metabolism in nutritional muscular dystrophy of chicks. J. Nutr..

[32] Yan Y.Q., Liu M., Xu Z.J., Xu Z.J., Huang Y.X., Li X.M., Chen C.J., Zuo G., Yang J.C., Lei X.G. (2024). Optimum doses and forms of selenium maintaining reproductive health via regulating homeostasis of gut microbiota and testicular redox, inflammation, cell proliferation, and apoptosis in roosters. J. Nutr..

[33] Zhao L., Liu M., Sun H., Yang J.C., Huang Y.X., Huang J.Q., Lei X., Sun L.H. (2023). Selenium deficiency-induced multiple tissue damage with dysregulation of immune and redox homeostasis in broiler chicks under heat stress. Sci. China Life Sci..

[34] Jo H., Kim B.G. (2023). Effects of dietary fiber in gestating sow diets—A review. Anim. Biosci..

[35] Li H., Ma L.T., Zhang L.L., Liu N.A., Li Z.Q., Zhang F., Liu X., Ma X.K. (2021). Dietary inulin regulated gut microbiota and improved neonatal health in a pregnant sow model. Front. Nutr..

[36] So S.R., Cherdthong A., Wanapat M. (2020). Improving sugarcane bagasse quality as ruminant feed with, cellulase, and molasses. J. Anim. Sci. Technol..

[37] Dai F.W., Lin T., Huang X., Shi X.L., Yang Y.J., Nong X., Zuo J.J., Liu H. (2023). Effects from supplementary feeding of bamboo powder in perinatal period on farrowing process, serum biochemical indexes, and fecal microbes of sows and offspring piglets. Front. Microbiol..

[38] Do S., Jang J.C., Lee G.I., Kim Y.Y. (2023). The role of dietary fiber in improving pig welfare. Animals.

[39] Qin F., Wei W.Y., Gao J.J., Jiang X.M., Che L.Q., Fang Z.F., Lin Y., Feng B., Zhuo Y., Hua L. (2023). Effect of dietary fiber on reproductive performance, intestinal microorganisms and immunity of the sow: A Review. Microorganisms.

[40] Li Y., Zhang L.J., Liu H.Y., Yang Y., He J.Q., Cao M., Yang M., Zhong W., Lin Y., Zhuo Y. (2019). Effects of the ratio of insoluble fiber to soluble fiber in gestation diets on sow performance and offspring intestinal development. Animals.

[41] Renteria-Flores J.A., Johnston L.J., Shurson G.C., Moser R.L., Webel S.K. (2008). Effect of soluble and insoluble dietary fiber on embryo survival and sow performance. J. Anim. Sci..

[42] Huang S.B., Cui Z.J., Hao X.Y., Cheng C.H., Chen J.Z., Wu D.Y., Luo H.F., Deng J.P., Tan C.Q. (2022). Dietary fibers with low hydration properties exacerbate diarrhea and impair intestinal health and nutrient digestibility in weaned piglets. J. Anim. Sci. Biotechnol..

[43] Guan Z.W., Yu E.Z., Feng Q. (2021). Soluble dietary fiber, one of the most important nutrients for the gut microbiota. Molecules.

[44] Zijlstra R.T., Jha R., Woodward A.D., Fouhse J., van Kempen T.A.T.G. (2012). Starch and fiber properties affect their kinetics of digestion and thereby digestive physiology in pigs. J. Anim. Sci..

[45] Li Y., Yang M., Zhang L.J., Mao Z.Y., Lin Y., Xu S.Y., Fang Z.F., Che L.Q., Feng B., Li J. (2022). Dietary fiber supplementation in gestating sow diet improved fetal growth and placental development and function through serotonin signaling pathway. Front. Vet. Sci..

[46] Liu P.Y., Wang Y.B., Yang G., Zhang Q.H., Meng L.B., Xin Y., Jiang X. (2021). The role of short-chain fatty acids in intestinal barrier function, inflammation, oxidative stress, and colonic carcinogenesis. Pharmacol. Res..

[47] Ndou S.P., Kiarie E., de Lange C.F., Nyachoti C.M. (2024). Interactive effects of dietary fiber and lipid types modulate the predicted production and absorption of cecal and colorectal short-chain fatty acids in growing pigs. J. Nutr..

[48] Igudesman D., Crandell J.L., Corbin K.D., Hooper J., Thomas J.M., Bulik C.M., Pence B.W., Pratley R.E., Kosorok M.R., Maahs D.M. (2023). Associations of dietary intake with the intestinal microbiota and short-chain fatty acids among young adults with type 1 diabetes and overweight or obesity. J. Nutr..

[49] Lammers-Jannink K.C.M., Pellikaan W.F., de Vries S., Stigter E.C.A., Gerrits W.J.J. (2023). Standardisation of the C:N ratio in ileal digesta changes relationships among fermentation end-products during in vitro hindgut fermentation in pigs. Animal.

[50] Jha R., Berrocoso J.F.D. (2016). Dietary fiber and protein fermentation in the intestine of swine and their interactive effects on gut health and on the environment: A review. Anim. Feed Sci. Technol..

[51] Wu Y., Liu X., Zou Y., Zhang X., Wang Z., Hu J., Han D., Zhao J., Dai Z., Wang J. (2024). Lactobacillus amylovorus promotes lactose utilization in small intestine and enhances intestinal barrier function in intrauterine growth restricted piglets. J. Nutr..

[52] Dong Z.L., Liu S., Deng Q.Q., Li G.Y., Tang Y.L., Wu X., Wan D., Yin Y.L. (2023). Role of iron in host-microbiota interaction and its effects on intestinal mucosal growth and immune plasticity in a piglet model. Sci. China Life Sci..

[53] Fan L., Xia Y., Wang Y., Han D., Liu Y., Li J., Fu J., Wang L., Gan Z., Liu B. (2023). Gut microbiota bridges dietary nutrients and host immunity. Sci. China Life Sci..

[54] Wang D.D., Tang G.F., Zhao L.C., Wang M.Y., Chen L.Y., Zhao C.C., Liang Z.Q., Chen J., Cao Y.C., Yao J.H. (2023). Potential roles of the rectum keystone microbiota in modulating the microbial community and growth performance in goat model. J. Anim. Sci. Biotechnol..

[55] Yu M., Gao T., Liu Z., Diao X.P. (2020). Effects of dietary supplementation with high fiber (Stevia Residue) on the fecal flora of pregnant sows. Animals.

[56] Tang X.P., Zhang K., Xiong K.N. (2022). Fecal microbial changes in response to finishing pigs directly fed with fermented feed. Front. Vet. Sci..

[57] Medawar E., Haange S.B., Rolle-Kampczyk U., Engelmann B., Dietrich A., Thieleking R., Wiegank C., Fries C., Horstmann A., Villringer A. (2021). Gut microbiota link dietary fiber intake and short-chain fatty acid metabolism with eating behavior. Transl. Psychiatry.

[58] Wang X.F., Tsai T.C., Deng F.L., Wei X.Y., Chai J.M., Knapp J., Apple J., Maxwell C.V., Lee J.A., Li Y. (2019). Longitudinal investigation of the swine gut microbiome from birth to market reveals stage and growth performance associated bacteria. Microbiome.

[59] Zhong J.G., Lan W.T., Feng Y.Q., Li Y.H., Shen Y.Y., Gong J.H., Zou Z., Hou X.H. (2023). Associations between dysbiosis gut microbiota and changes of neurotransmitters and short-chain fatty acids in valproic acid model rats. Front. Physiol..

[60] Jeong J.J., Jin Y.J., Ganesan R., Park H.J., Min B.H., Jeong M.K., Yoon S.J., Choi M.R., Sharma S.P., Jang Y.J. (2024). Multistrain probiotics alleviate diarrhea by modulating microbiome-derived metabolites and serotonin pathway. Probiotics Antimicrob. Proteins.

